# Machine learning prediction and interpretability analysis of high-risk chest pain: a study from the MIMIC-IV database

**DOI:** 10.3389/fphys.2025.1594277

**Published:** 2025-06-30

**Authors:** Hongyi Chen, Haiyang Song, Hongyu Huang, Xiaojun Fang, Huang Chen, Qingqing Yang, Junyu Zhang, Wenjun Ding, Zheng Gong, Jun Ke

**Affiliations:** ^1^ Fujian Provincial Hospital, Department of Emergency, Fuzhou, China; ^2^ Shengli Clinical Medical College of Fujian Medical University, Fuzhou, China; ^3^ Fujian Funeng General Hospital, Department of Emergency, Fuzhou, China; ^4^ Fuzhou University Affiliated Provincial Hospital, Department of Emergency, Fuzhou, China; ^5^ Fujian Provincial Key Laboratory of Emergency Medicine, Fujian Provincial Hospital, Fuzhou, China; ^6^ School of Electronics and Information Engineering, Guangxi Normal University, Guilin, China; ^7^ School of Informatics, Xiamen University, Xiamen, China

**Keywords:** bayesian optimization, model interpretability, high-risk chest pain prediction, MIMIC-IV, machine learning (ML)

## Abstract

**Background:**

High-risk chest pain is a critical presentation in emergency departments, frequently indicative of life-threatening cardiopulmonary conditions. Rapid and accurate diagnosis is pivotal for improving patient survival rates.

**Methods:**

We developed a machine learning prediction model using the MIMIC-IV database (n = 14,716 patients, including 1,302 high-risk cases). To address class imbalance, we implemented feature engineering with SMOTE and under-sampling techniques. Model optimization was performed via Bayesian hyperparameter tuning. Seven algorithms were evaluated: Logistic Regression, Random Forest, SVM, XGBoost, LightGBM, TabTransformer, and TabNet.

**Results:**

The LightGBM model demonstrated superior performance with accuracy = 0.95, precision = 0.95, recall = 0.95, and F1-score = 0.94. SHAP analysis revealed maximum troponin and creatine kinase-MB levels as the top predictive features.

**Conclusion:**

Our optimized LightGBM model provides clinically significant predictive capability for high-risk chest pain, offering emergency physicians a decision-support tool to enhance diagnostic accuracy and patient outcomes.

## Introduction

High-risk chest pain represents a subset of chest pain presentations that are associated with a high probability of life-threatening conditions such as acute coronary syndrome (ACS), pulmonary embolism (PE), or aortic dissection. Although widely used in clinical practice, the term “high-risk chest pain” lacks a universally accepted definition and may vary depending on institutional protocols or clinician judgment. In general, high-risk cases are identified based on clinical features such as ongoing or recurrent chest pain, dynamic electrocardiographic changes, hemodynamic instability, a history of coronary artery disease, or elevated cardiac biomarkers (Amsterdam et al., 2014; Backu et al., 2013). Risk stratification tools—such as the HEART score, TIMI score, and GRACE score—are often employed to aid in identifying patients at elevated risk of adverse cardiac events (Six et al., 2008; Antman et al., 2000; Granger et al., 2003).

In emergency settings, however, the diagnostic process is frequently complicated by the heterogeneous and often nonspecific nature of chest pain symptoms. Additionally, the absence of definitive early indicators can make it challenging to differentiate high-risk conditions from benign causes. Clinician experience and subjective interpretation of symptoms, ECG findings, and clinical history often play a significant role, which may inadvertently contribute to misdiagnosis or delayed treatment (Rohacek et al., 2012). Therefore, accurate and timely identification of high-risk chest pain remains essential to reduce mortality and improve clinical outcomes.

In recent years, machine learning (ML) has emerged as a promising tool in clinical decision support systems, offering the ability to uncover complex patterns from large-scale medical data. Despite this progress, existing research on ML-based prediction models for high-risk chest pain remains limited in several key aspects. First, many studies have not fully addressed the issue of class imbalance, which can severely degrade model performance on rare but critical outcomes. Second, the comparative performance of advanced ML models specifically tailored for structured medical data, such as TabTransformer and TabNet, has not been systematically evaluated in this context. Third, few studies have leveraged interpretability techniques like SHAP to provide clinically meaningful insights into model predictions.

To bridge these gaps, this study develops a robust ML-based prediction model for high-risk chest pain using the publicly available MIMIC-IV database. The main contributions of this paper are as follows:

1. A comprehensive machine learning pipeline was developed, incorporating feature engineering, Synthetic Minority Over-sampling Technique (SMOTE), random under-sampling, and Bayesian hyperparameter optimization to address class imbalance and enhance model performance.
2. A comparative evaluation of multiple classical and state-of-the-art machine learning algorithms, including Logistic Regression, Random Forest, SVM, XGBoost, LightGBM, TabTransformer, and TabNet, was conducted on a large clinical dataset.
3. The LightGBM model was identified as the best-performing model, achieving outstanding accuracy (0.95), precision (0.95), recall (0.95), and F1 score (0.94).
4. SHAP interpretability analysis was used to uncover the most influential clinical features, with maximum troponin and creatine kinase MB emerging as key predictors of high-risk chest pain.

The remainder of the paper is organized as follows: Section 2 provides a comprehensive review of background and related literature, emphasizing the clinical importance of accurately identifying high-risk chest pain and summarizing current machine learning applications in emergency diagnosis. Section 3 outlines the methodological framework of the study, covering the overall experimental design, model development strategies, and techniques for enhancing both predictive performance and interpretability. Section 4 presents the experimental results, compares the performance of various models, and discusses the clinical relevance and implications of the findings. Section 5 concludes the study by summarizing the main contributions, discussing its limitations, and proposing future research directions to further enhance model performance and support real-world clinical application.

## Background and related literature

Chest pain is one of the most common complaints in the emergency department and often indicates a potentially life-threatening condition, such as acute coronary syndrome, pulmonary embolism, and aortic dissection. These high-risk diseases have a high mortality rate and can lead to serious consequences if not diagnosed and treated in time. However, when dealing with patients with chest pain, clinicians need to not only quickly identify risk factors, but also avoid the waste of resources and burden on patients caused by over-examination. How to balance the early identification of high-risk chest pain and the reasonable allocation of medical resources has become an urgent problem for medical science.

In recent years, the popularity of electronic health record data has provided important support for risk assessment and prediction of high-risk chest pain. EHR data not only contains basic demographic information of patients, but also records a large number of clinical examination results and treatment processes. By mining these data, data-driven models can be built to assist doctors in early diagnosis and risk prediction, thereby improving emergency efficiency and diagnostic accuracy.

TIMI and HEART score are the traditional tools for chest pain risk assessment, which have been widely validated, but these experience-based scoring systems are still subject to subjectivity and lack of sensitivity and specificity. With the advancement of artificial intelligence technology, machine learning (ML) has shown great potential in chest pain diagnosis and risk prediction due to its ability to process complex non-linear data. Numerous studies have applied ML to the risk assessment and diagnosis of emergency chest pain, not only optimizing the performance of existing tools, but also showing superior performance to traditional methods in clinical practice. Zhang et al. (Wang et al., 2020) developed an ANN-based model that used patient clinical, demographic, and laboratory data to predict AMI and 30-day mortality, with an AUC of 0.907 and 0.888, respectively, significantly better than traditional methods. Wu et al. (2019) predicted MACE within 90 days through RF model combined with invasive and non-invasive variables, and the AUC reached 0.853, higher than the HEART score. The MI3 model proposed by Than et al. (2019) combined with SVM algorithm to predict AMI has an AUC of up to 0.963 and provides excellent sensitivity and specificity at different risk thresholds.

In addition to traditional machine learning techniques, recent research has shown increasing interest in combining deep learning models such as convolutional neural networks (CNNs), recurrent neural networks (RNNs), and long short-term memory (LSTM) networks with ensemble methods like XGBoost, optimized using metaheuristic algorithms. These hybrid and optimization-enhanced models are particularly effective for medical applications involving high-dimensional, imbalanced, or noisy data. For instance, Kumar and Hasija, (2023) developed a hybrid CNN-XGBoost model optimized by a modified arithmetic optimization algorithm for early COVID-19 diagnosis from chest X-rays, achieving high accuracy under data imbalance. Gupta and Hasija, (2023) applied CNNs with boosting algorithms tuned by metaheuristics for classifying respiratory conditions using audio signals. In the neurological domain, Sharma et al. (2023) used LSTM models optimized by metaheuristics for detecting Parkinson’s disease from gait time series. Similar frameworks have been proposed for respiratory disease detection from audio (Hasija and Kumar, 2023) and anomaly detection in ECG signals (Ali and Hasija, 2023), demonstrating the versatility and efficacy of such AI models in diverse clinical scenarios. These advances highlight the potential for translating such methods to high-risk chest pain assessment, where interpretability and predictive reliability are critical.

To overcome potential algorithmic bias and lack of transparency in healthcare, a large number of XAI approaches have recently been investigated. These methods can be grouped into three categories based on interpretation, implementation, and model dependency levels. Interpretation of the model can be done both locally and globally. The local level explains the model decisions of a single instance, while the global level explains the model’s entire decisions. The implementation level is further divided into internal interpretability and post-interpretability. Intrinsic explainability refers to a model that is considered explainable due to its simple architecture (e.g., TabNet). Post-interpretability refers to the application of interpretative methods (e.g., LIME (Ribeiro et al., 2016) and SHAP) after model training. The model dependency standard deals with both model-specific and model-independent interpreters. Model-specific methods are limited to explaining specific types of algorithms. Although the goal of model transparency is established, these methods cannot be used for any model without re-changing its interpretation mechanism (Alicioglu and Sun, 2022).

Unlike model-specific interpreters, model-independent methods receive more attention for their ability to be applied and tested on any “white box or black box” model. The general idea is to explain and explain the decisions behind the model’s output. A useful and popular contribution to model-independent XAI is SHAP. SHAP (Lundberg et al., 2020; Shapley and Roth, 1988) is a Shapley value determination method based on cooperative game theory, whose core goal is to calculate the impact of each feature on instance prediction. Based on this, Gu et al. (2020) used different feature weights of the variables (i.e., Shapley values) to interpret the positive and negative results of the breast cancer recurrence classification. This is an example of how SHAP provides local interpretability. You can also aggregate the Shapley values of all instances in the sample to calculate the global feature importance score. Researchers use feature importance to help explain features between features and the results generated by the model. For example, Meena and Hasija, (2022) used feature weights to sequence and identify important genes found to be associated with the progression of squamous cell carcinoma. Thus, what makes SHAP so reliable is that it takes into account all possible predictions for the instances in the sample using all possible combinations of inputs. This enables it to guarantee features such as consistency and local accuracy. On the downside, Shapley values take a long time to calculate and are therefore an exhaustive method.

Therefore, after comprehensive consideration, this study proposed a machine learning-based prediction model for high-risk chest pain based on a widely used clinical data set, MIMIIC-IV. We will focus on exploring the process of feature engineering, missing value processing, model training, and Bayesian optimization tuning, while using model-independent SHAP for global interpretation to help clinicians better understand and apply the predicted results, and hopefully provide a reference for future data-based risk assessment methods.

## Materials and methods

**FIGURE 1:**
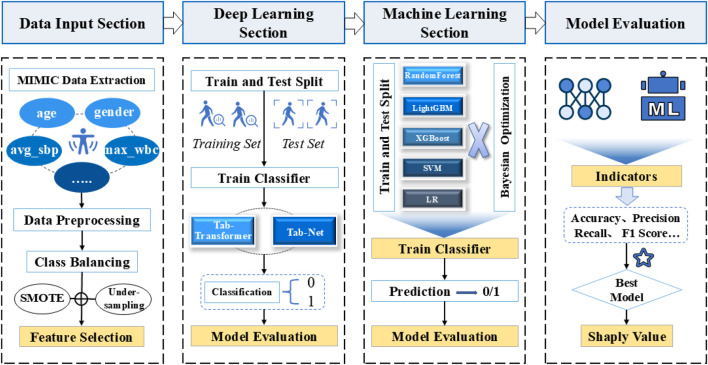
General frame diagram.

**FIGURE 2:**
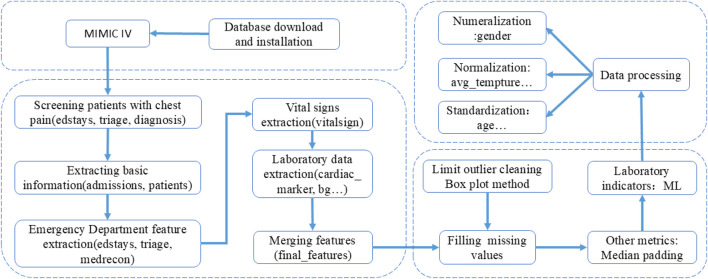
Flow chart of feature extraction.

The overall method of the experiment is shown in Figure 1, and the process of database feature extraction is shown in Figure 2.

### Model selection

In the high-risk chest pain prediction task, choosing the right machine learning model is crucial. The task involves extracting effective information from clinical data to help physicians quickly identify high-risk patients. To comprehensively evaluate the performance of different models, we chose a variety of classical and modern machine learning methods to compare. These models include traditional machine learning algorithms (such as logistic regression, random forest, support vector Machine SVM), ensemble learning algorithms (such as XGBoost and LightGBM), and deep learning models (such as TabTransformer and TabNet). Some popular models such as AdaBoost, CatBoost, and Extreme Learning Machine (ELM) were not included in this study, and this decision was made based on both methodological and practical considerations. AdaBoost, although historically important, is sensitive to noise and outliers, which are common in real-world clinical data. Its performance tends to lag behind more advanced boosting methods such as XGBoost and LightGBM, particularly in large-scale or imbalanced settings [4]. CatBoost is highly effective for high-cardinality categorical features, but given that our dataset contained mostly preprocessed or low-cardinality categorical variables, its advantages would not be fully utilized. Moreover, CatBoost can be computationally more demanding when extensively tuned. As for ELM, despite its extremely fast training, it lacks robustness and generalization capability for complex, high-dimensional data like EHRs and does not support interpretability tools or native handling of missing values, limiting its applicability in clinical settings (Huang et al., 2006).

Logistic regression. Logistic regression is a basic linear model, which is widely used in binary classification problems. It makes predictions by weighted summing features and converting the results into probabilities via the Sigmoid function. In the medical field, logistic regression has a good interpretable ability and can intuitively reveal the influence of various features on the predicted results (Kim et al., 2020). However, logistic regression is mainly suitable for situations where there is a linear relationship between features, so it may not perform as well as other more complex models when faced with complex nonlinear relationships.

Random Forest. A random forest is an ensemble learning method that makes predictions by training multiple decision trees and voting on the results. Its advantages lie in its ability to process high-dimensional data without easy overfitting, automatic feature selection, and certain robustness to missing data (Breiman, 2001). In chest pain prediction tasks, random forest can better capture the nonlinear relationship between features, but its “black box” nature makes it less interpretable, which may be a limitation for medical scenarios (Li et al., 2021; Wang et al., 2023).

SVM. SVM is a powerful classification model that separates different classes of data by finding an optimal hyperplane. SVM performs well in high-dimensional data and can effectively handle complex nonlinear classification problems (Zhou et al., 2022). However, the disadvantage of SVM is that the training time is long, especially when the data volume is large, and the selection of parameters is more sensitive, which may affect the stability and generalization ability of the model.

XGBoost. XGBoost (Extreme Gradient Boosting) is an ensemble learning method based on gradient lifting trees that minimizes the loss function by gradually adjusting the weights of weak classifiers. It has high efficiency in processing large-scale data, and can automatically process categorical features and missing data (Chen and Guestrin, 2016). In medical datasets, especially those with complex feature interactions, XGBoost can capture these complex non-linear relationships and make accurate predictions (Li et al., 2022). This ability makes XGBoost particularly effective in high-risk chest pain prediction tasks, extracting critical information from patients’ clinical characteristics to support rapid diagnosis.

LightGBM. LightGBM (Light Gradient Boosting Machine) is a machine learning algorithm based on gradient boosting that shows significant computational efficiency advantages when dealing with large-scale data sets. Compared with the traditional gradient lifting algorithm, LightGBM adopts a leaf-based splitting strategy and uses histogram algorithm to speed up the calculation process, which makes it effective in reducing memory consumption and speeding up the training speed when processing high-dimensional data (Ke et al., 2017). LightGBM has demonstrated its capabilities in the task of classifying medical data, especially in situations where data is imbalanced. Data imbalance problems are more common in medical classification tasks, for example, the proportion of patients with high-risk chest pain tends to be low, which makes it more difficult for the model to correctly predict a small number of classes. Traditional machine learning models tend to perform poorly in such situations, tending to be biased toward predicting most classes, resulting in lower recall rates for a few classes. However, LightGBM, through its built-in sample weight adjustment mechanism and the ability to support custom loss functions, has shown significant advantages in dealing with unbalanced data (Xu et al., 2021; He et al., 2020; Zhang et al., 2021).

TabTransformer. TabTransformer is a deep learning model applicable to tabular data. It uses self-attention mechanism to capture complex interactive relationships among category features (Huang et al., 2020). Compared to traditional models, TabTransformer has a strong capability in feature interaction modeling, especially suitable for high-dimensional data containing categorical features. The model uses deep neural networks combined with attention mechanisms to automatically learn complex patterns in the data. However, TabTransformer typically requires large computing resources and takes a long time to train.

TabNet. TabNet is a tabular data processing model based on deep learning, which combines the advantages of neural network and decision tree to improve the prediction accuracy of the model (Arik and Pfister, 2021). TabNet shows good performance when dealing with large scale and sparse data, and can provide certain interpretability. Nevertheless, TabNet’s training time and computing resource consumption are large and may not be the best choice for resource-limited environments.

### Bayesian optimization (BO)

Trial-and-error hyperparameter tuning is tedious and often leads to unsatisfactory results (Massaoudi et al., 2021). Therefore, robust tuning methods are essential, especially when the goal of optimization is to find the maximum value of an unknown function at the sampling point (Equation 1), as in (Shi et al., 2021):

$$p^{+}=\arg\underset{p\in ⊘}{\max}\vartheta \left(p\right)$$ (1)

Where p represents the sampling point, ⊘ represents the search space of the sampling point p, ϑ represents the unknown objective function, and $p^{+}$ represents the location where the unknown objective function is largest.

Compared with commonly used GS and RS technologies, BO is an efficient hyperparameter optimization algorithm (Mockus and Marchuk, 1975). In GS and RS, each evaluation in its iteration is independent of the previous evaluation, which increases the waste of time in evaluating poorly performing areas of the hyperparameter search space. This problem is solved by BO, which combines the prior information of ϑ with the sampling points, approximates the posterior distribution of the objective function through Bayes’ theorem (Eggensperger et al., 2013), and then uses the posterior information to evaluate the global optimal value.

The two main steps involved in executing BO are as follows (Kulshrestha et al., 2020):

(1) BO algorithm tries to fit the proxy function by randomly selecting several data points on ϑ . Due to the high flexibility, robustness, accuracy, and analysis traceability of Gaussian processes (GP) (Martinez-Cantin, 2017), this study uses GP to update the proxy function to form a posterior distribution of ϑ .
(2) The posterior distribution formed in step 1 is used to create a collection function that explores new regions in the search space and uses the known regions to get the best results (Injadat et al., 2018). The exploration and development process continues, and the agent model is updated with new results until predefined stop criteria are met. The criterion for locating the next sampling point is to maximize the collection function. In this paper, expected improvement (EI) (Cheng et al., 2019) is used as the collection function.

### Interpretability

In order to improve the interpretability of the model, this study used SHAP to determine the influence, dependence, and interaction of global features on the classification of high-risk chest pain from sugar (Lundberg et al., 2020; Shapley and Roth, 1988). SHAP uses the principles of cooperative game theory to assign each input feature an importance score for a given prediction. Game theory has a set of rules, players in the game have a set of strategies and some kind of reward, and the Shapley value is used to reveal each player’s contribution to the game. To explain this model, the policy represents the outcome of the program, the actor represents the feature, and the reward is the quality of the outcome obtained. Here, the Shapley value reveals the contribution of a given feature to the overall prediction, and the sampling process can be repeated to improve the approximation of the marginal contribution. The SHAP value can then be defined as the weighted average of the marginal contributions of all possible alliances—F— ! expressed as (Lundberg et al., 2020):

$$\psi_{i}\left(f\right)=\underset{\left\{S\subseteq F\right\}\backslash \left\{i\right\}}{\sum }\frac{|S|!\;\left(|F|-|S|-1\right)!}{|F|!}\cdot \left[f\left(x_{S\cup \left\{i\right\}}\right)-f\left(x_{S}\right)\right]$$ (2)

In the above formula, is the weighted average of the Shapley values provided by feature i in the above federation of all excluded functions, F is the total number of features, and S is a subset of F, predicting for models using feature i and predicting for models not using feature i.

Compared with lime, this calculation (Equation 2) increases the time complexity of SHAP. However, SHAP uses all subsets of the input data, which provides better local accuracy and consistency compared to lime.

### DataSet

The MIMIC-IV database collects detailed clinical data on ICU patients in the Boston area from 2008 to 2019, including patient demographic and hospitalization information, physiological monitoring, laboratory tests, medication, diagnostic codes, and other information, which can be used for clinical analysis of high-risk chest pain. The author obtained permission to use the database (record ID: 1,3992078) after completing the CIT1 project training. Use structured query language (SQL) and PostgreSQL13 to extract data.

### Data preprocessing

Feature selection. To construct a clinically meaningful and interpretable predictive model for high-risk chest pain, we extracted four categories of variables from the MIMIC-IV database. The selection of these variables was informed by clinical guidelines, prior literature on acute coronary syndrome (ACS) risk stratification, and expert consultation with emergency physicians and cardiologists. This approach ensured that the features used were both data-available and clinically relevant for identifying patients at elevated risk of adverse cardiovascular outcomes.

The first category includes demographic and hospitalization information, such as patient ID, age, gender, length of stay, and type of admission. Age and gender are well-established risk factors in cardiovascular disease prognosis, and admission type often reflects the acuity of the patient’s condition at presentation.

The second category comprises emergency-related variables, including emergency department (ED) length of stay, chief complaint, triage acuity level (Triage_acuity) , and medications administered during the ED visit. These features provide contextual insight into the severity of the presenting symptoms, early clinical impressions, and initial treatment decisions, all of which are associated with short-term outcomes in chest pain patients.

The third category includes vital signs, such as body temperature, heart rate, respiratory rate, oxygen saturation, and blood pressure (systolic, diastolic, and mean arterial pressure), recorded as maximum, minimum, and average values. These are critical indicators of hemodynamic stability and are routinely used in early warning systems and risk scores such as the HEART and TIMI scores (Backu et al., 2013; Antman et al., 2000).

The fourth category encompasses laboratory biomarkers, including the maximum recorded values of troponin, creatine kinase-MB (CK-MB), sodium, potassium, white blood cell (WBC) count, C-reactive protein (CRP), and lactic acid. Among these, troponin and CK-MB are of particular importance. According to the Fourth Universal Definition of Myocardial Infarction, cardiac troponins are the gold-standard biomarkers for detecting myocardial injury and diagnosing acute coronary syndromes (Thygesen et al., 2018). CK-MB, though less specific than troponin, remains clinically valuable in certain settings, especially where high-sensitivity troponin assays are not available or for assessing reinfarction (Thygesen et al., 2018; Newby and Granger, 2012). Additionally, CRP, WBC, and lactate provide insights into systemic inflammation and tissue hypoperfusion, both of which are prognostically important in acute cardiovascular and septic conditions (Tang et al., 2020).

In total, 38 clinically and statistically relevant features were constructed from 40,438 clinical records. After applying inclusion criteria and data cleaning, a final cohort of 14,716 patients was included in the study, of whom 1,302 (8.84%) were identified as having high-risk chest pain. This feature selection process ensures the model’s alignment with clinical practice and enhances its potential utility for real-time risk prediction in emergency care settings. Table 1 shows baseline information for all patients in the MIME-IV database.

**TABLE 1:** Comparison of baseline characteristics in the Low Risk and High Risk groups.

Variables	Total (n = 14,716)	Low risk	High risk	P-value
gender (*No units*)	-	-	-	<0.001
Male (*No units*)	7864 (53.5%)	7119 (53.1%)	745 (42.7%)	-
Female (*No units*)	6852 (46.5%)	6215 (46.9%)	557 (57.3%)	-
age (*years*)	61.03 (50.00, 73.00)	60.86 (50.00, 73.00)	62.78 (52.00, 75.00)	<0.001
avg_temperature (*°F*)	98.07 (97.84, 98.30)	98.07 (97.85, 98.30)	98.02 (97.83, 98.20)	<0.001
avg_heartrate (*bpm*)	78.95 (68.00, 88.33)	78.25 (67.67, 87.67)	86.19 (71.00, 99.91)	<0.001
avg_resprate (*bpm*)	17.73 (16.56, 18.67)	17.64 (16.50, 18.50)	18.68 (17.00, 19.89)	<0.001
avg_O2sat (*%*)	97.68 (96.67, 99.00)	97.69 (96.67, 99.00)	97.58 (96.60, 99.00)	0.015
avg_sbp (*mmHg*)	130.05 (117.00, 141.50)	130.64 (117.56, 142.00)	123.93 (111.38, 134.26)	<0.001
avg_dbp (*mmHg*)	72.21 (64.50, 79.67)	72.32 (64.62, 79.75)	71.10 (62.88, 78.63)	<0.001
temperature_range (*°F*)	0.54 (0.10, 0.89)	0.54 (0.10, 0.90)	0.53 (0.00, 0.89)	0.611
heartrate_range (*bpm*)	15.00 (7.00, 21.00)	14.66 (7.00, 20.00)	18.50 (9.00, 23.51)	<0.001
resprate_range (*bpm*)	4.69 (2.00, 7.00)	4.55 (2.00, 6.00)	6.18 (4.00, 8.00)	<0.001
O2sat_range (*%*)	2.82 (1.00, 4.00)	2.77 (1.00, 4.00)	3.32 (1.00, 5.00)	<0.001
sbp_range (*mmHg*)	27.62 (15.00, 38.00)	27.49 (15.00, 38.00)	28.97 (17.00, 39.00)	0.002
dbp_range (*mmHg*)	21.53 (12.00, 30.00)	21.33 (12.00, 30.00)	23.62 (14.00, 31.00)	<0.001
max_troponin (*ng/mL*)	0.39 (0.27, 0.40)	0.34 (0.27, 0.39)	0.89 (0.30, 1.23)	<0.001
max_ckmb (*ng/mL*)	6.12 (3.97, 6.23)	5.55 (3.00, 6.09)	12.09 (4.00, 13.00)	<0.001
max_sodium (*mmol/L*)	140.78 (139.00, 143.00)	140.76 (139.00, 143.00)	140.94 (139.24, 142.00)	0.043
max_potassium (*mmol/L*)	4.43 (4.10, 4.70)	4.42 (4.10, 4.70)	4.53 (4.20, 4.80)	<0.001
max_wbc ( $10^{9}$ */L*)	8.64 (6.40, 10.20)	8.49 (6.30, 10.00)	10.24 (7.40, 12.20)	<0.001
max_lactate (*mmol/L*)	2.20 (2.04, 2.31)	2.18 (2.04, 2.30)	2.38 (2.10, 2.41)	<0.001

The table compares the baseline characteristics between the Low Risk and High Risk groups. In each group, continuous variables are described using the median and the first and third quantiles, formatted as M $\left(Q_{1},Q_{3}\right)$ , and categorical variables are described using counts and proportions, formatted as n (%).

**FIGURE 3:**
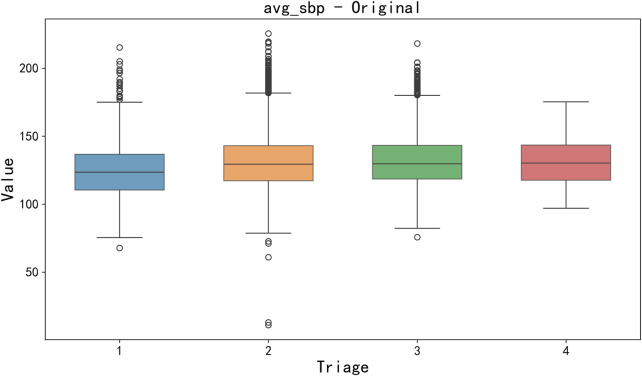
Box plot of avg_sbp raw data.

**FIGURE 4:**
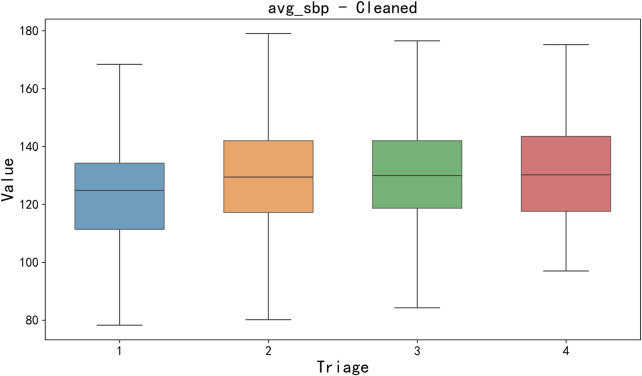
Box diagram of avg_sbp cleaned data.

Data cleaning and missing value processing. Firstly, a key step of data preprocessing is the detection and processing of outliers. In order to detect outliers in the data, we adopted the boxplot method. Boxplots identify outliers by five generalizations of the visualized data (minimum, lower quartile Q_1_, median Q_2_, upper quartile Q_3_, and maximum). Specifically, an outlier is defined as a value 1.5 times less than the lower quartile (Q_1_) quartile (IQR) or 1.5 times more than the upper quartile (Q_3_). After detecting outliers, we choose to exclude outliers that are obvious in some features (such as clinical measurement errors or extreme data points) to avoid having a negative impact on the training and prediction of the model. For the sake of brevity, only box plots of mean body temperature and mean systolic pressure data are shown below, as shown in Figures 3, 4.

Secondly, the handling of missing values can significantly influence the outcomes of subsequent experiments. For features with relatively low proportions of missing data, missing values were imputed using the mean. For features with higher rates of missingness—such as max_troponin, max_ckmb, max_sodium, max_potassium, max_wbc, and max_lactate—four machine learning models were employed for imputation: XGBoost, Random Forest, Ridge Regression, and LightGBM. To determine the most effective imputation method, Bayesian optimization was conducted for hyperparameter tuning of each model, with Mean Squared Error (MSE) used as the evaluation metric. The experimental results are summarized in Table 2.

**TABLE 2:** MSE for different models on various targets.

Target	XGBoost	Random forest	Ridge regression	LightGBM
max_troponin (*ng/mL*)	0.46	0.44	0.49	0.43
max_ckmb (*ng/mL*)	44.41	44.93	52.17	44.47
max_sodium (*mmol/L*)	8.91	8.93	8.93	8.91
max_potassium (*mmol/L*)	0.23	0.23	0.23	0.23
max_wbc ( $10^{9}$ */L*)	9.29	9.33	9.37	9.29
max_lactate (*mmol/L*)	1.41	1.42	1.37	1.38

Experimental results indicated that different models exhibited varying performance across different features. For example, XGBoost yielded the lowest Mean Squared Error (MSE) for imputing max_sodium and max_ckmb, whereas LightGBM demonstrated superior performance for imputing max_potassium, max_troponin, and max_wbc. Consequently, the imputation of each feature was carried out using the model that achieved the lowest MSE.

**FIGURE 5:**
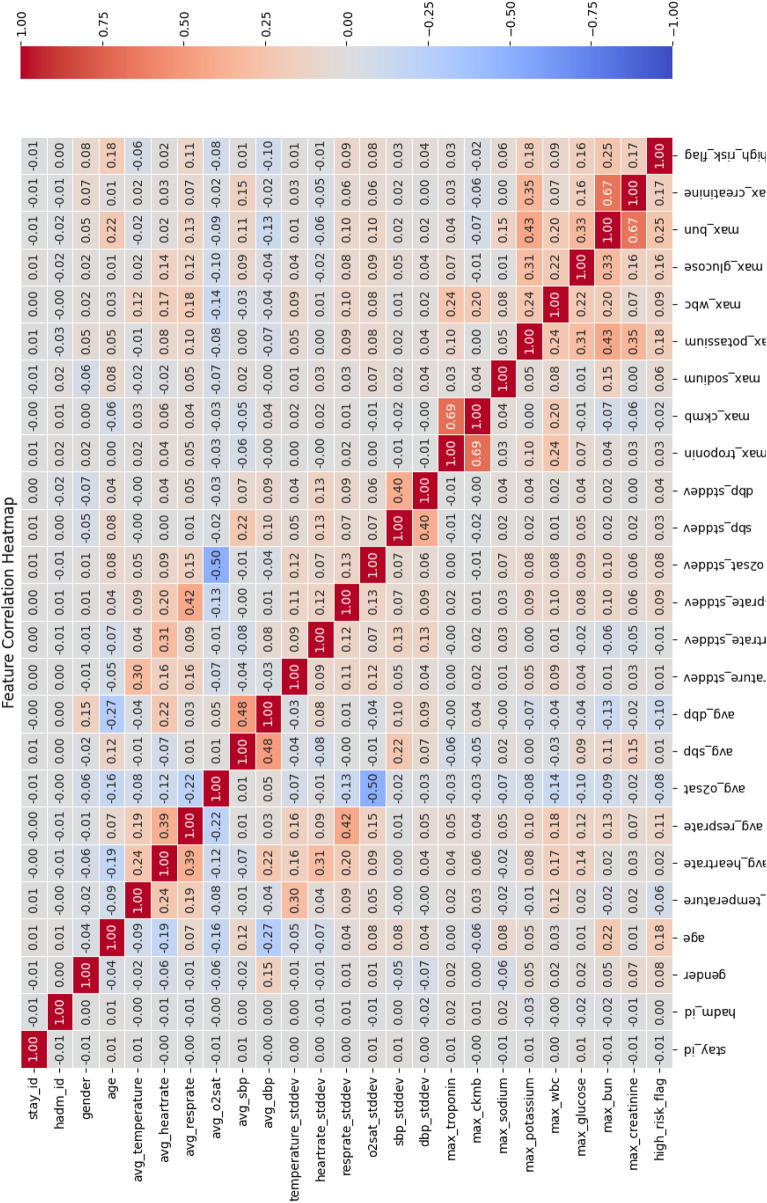
Feature correlation heat map.

Correlation analysis between feature and target variable. In this study, the input features included multiple physiological and clinical data, and we used the Pearson Correlation Coefficient to quantify the linear relationship between each feature and the target variable (“high risk” or “low risk”) for predicting chest pain risk. The heatmap of the correlation matrix was used for visualization, as shown in Figure 5.

The correlation matrix calculated using the Pearson correlation coefficient shows that there is a significant linear relationship between multiple features and the target variable of chest pain risk. Specifically, the following features showed strong correlations:

• max_troponin (troponin concentration) and max_ckmb (CK-MB level): The Pearson correlation coefficient between these two features and the target variable was 0.69, indicating a strong positive correlation with the occurrence of high-risk chest pain. This is consistent with existing clinical studies that troponin is often used as a marker of heart injury and can effectively predict high-risk patients (Michael et al., 2022).
•max_bun and max_creatinine: The correlation coefficient between them and the target variable is 0.67, indicating a strong positive correlation with the high-risk flag.

In addition, certain features—such as hadm_id and avg_O2sat—were observed to have low correlation with the target variable, suggesting limited contribution to the prediction task. As a result, these features were considered for removal to simplify the model structure and enhance computational efficiency.

Data normalization and standardization. After the aforementioned preprocessing steps, a total of 14 features were selected as input variables, and one feature (high_risk_flag) was designated as the target variable, comprising 14,717 data points. Prior to model training, two scaling methods—standardization and normalization—were applied to different types of features (Ramadhan et al., 2024). For features exhibiting Gaussian or near-Gaussian distributions (e.g., max_troponin, max_ckmb, max_sodium), standardization was employed to improve model convergence and minimize inter-feature influence due to large numerical ranges. In contrast, normalization was applied to features with known bounded ranges and relatively small variation (e.g., age, heart_rate), scaling them to the [0, one] interval to ensure consistency in scale and reduce feature disparities.

### Experimental environment

The experimental environment of this paper is shown in Table 3.

**TABLE 3:** Experimental environment.

Name	Configuration information
Processor	i7-13620H
Graphics card	RTX 4060Ti (24G)
Programming language	Python 3.9
Archive	Postgres 13
Operating system	Windows 10
Programming platform	Pycharm 2020 Community

### Evaluation index

The ROC curve is a relationship graph of TPR (Sensitivity) and FPR (1-Specificity) for diagnostic specificity, and AUC summarizes the accuracy of the model (Mandrekar, 2010). The ROC AUC indicator is in the range [0,1], where 0 indicates completely inaccurate results, 0.5 indicates that the classifier cannot distinguish between positive and negative category results, 0.7–0.8 is acceptable, 0.8–0.9 is considered excellent, and >0.9 is considered outstanding (Hosmer et al., 2013).

These indicators are calculated by the following formula (Equations 3–8):

$$Acc=\frac{TP+TN}{TP+TN+FP+FN}$$ (3)

$$Precision=\frac{TP}{TP+FP}$$ (4)

$$Recall=\frac{TP}{TP+FN}$$ (5)

$$F1\;Score=2\times \frac{Precision\times Recall}{Precision+Recall}$$ (6)

$$FPR=\frac{FP}{FP+TN}$$ (7)

$$AUC=\int_{0}^{1}Recall\left(FPR\right)\;dFPR$$ (8)

Cohen’s Kappa (κ) is a measure that compares the accuracy of an observation to the expected accuracy (Equation 9). The systematic interpretation of κ is as follows (Viera et al., 2005):

•If κ<0 , the performance is poor.
• κ=0.01−0.20 , the consistency was slightly better.
• κ=0.21−0.40 , fair and consistent.
• κ=0.61−0.80 , sustainable.
• κ=0.81−0.99 , almost identical.

$$\kappa =\frac{P_{0}-P_{e}}{1-P_{e}}$$ (9)

## Results and discussion

### Experimental result

Accuracy, precision, recall, specificity, and F1 score are commonly used metrics for classification problems (Ozkok and Celik, 2022). In addition, alternative metrics such as NPV and PPV are added. The higher the value of these indicators, the more preferred the model. Table 4 summarizes the performance of all models on both the independent test set and via 5-fold stratified cross-validation. As shown in Table 4, LightGBM achieved the highest test AUC (0.89, 95% CI [0.878–0.902]), Accuracy (0.95) and macro-F1 (0.94), followed closely by XGBoost and TabTransformer. In terms of generalization, LightGBM maintained the best 5-fold CV AUC (0.885 ± 0.010), suggesting robust discriminative ability across partitions. SVM and Logistic Regression showed relatively lower performance with wider AUC confidence intervals and larger variance in cross-validation. These results demonstrate the advantage of modern boosting and attention-based models in high-risk chest pain prediction.

**TABLE 4:** Summary of model performance on test and cross-validation sets.

Models	AUC	95% CI	Accuracy	Precision	Recall	F1 score	PPV	NPV	AUC (5-CV ± SD)
Random Forest	0.85	[0.837–0.863]	0.91	0.92	0.91	0.92	0.91	0.90	0.848 ± 0.012
LightGBM	0.89	[0.878–0.902]	0.95	0.95	0.95	0.94	0.95	0.94	0.885 ± 0.010
XGBoost	0.87	[0.860–0.880]	0.94	0.94	0.95	0.94	0.94	0.93	0.872 ± 0.011
SVM	0.77	[0.759–0.783]	0.80	0.89	0.80	0.84	0.82	0.78	0.768 ± 0.015
Logistic Regression	0.73	[0.718–0.743]	0.72	0.88	0.72	0.78	0.76	0.70	0.725 ± 0.017
TabTransformer	0.80	[0.788–0.813]	0.85	0.84	0.88	0.85	0.85	0.83	0.801 ± 0.013
TabNet	0.77	[0.759–0.785]	0.87	0.90	0.87	0.88	0.88	0.85	0.775 ± 0.012

Pairwise DeLong tests were performed to evaluate the statistical significance of differences in AUC between models, as presented in Table 5. The results indicate that LightGBM achieved significantly better discriminative performance compared to Logistic Regression ( p < 0.0001) and SVM ( p = 0.0189). However, no statistically significant difference was observed between LightGBM and XGBoost ( p = 0.3841) or TabNet ( p = 0.1202), suggesting comparable AUC values among top-performing models.

**TABLE 5:** DeLong test p -values between model pairs. Bold p-values below 0.05 indicate statistically significant differences.

Models	Random forest	LightGBM	XGBoost	SVM	Logistic regression	TabTransformer	TabNet
Random Forest	1.0000	0.0412	0.2173	0.0305	0.0008	0.0894	0.0527
LightGBM	0.0412	1.0000	0.3841	0.0189	<0.0001	0.1465	0.1202
XGBoost	0.2173	0.3841	1.0000	0.0628	0.0023	0.2081	0.1806
SVM	0.0305	0.0189	0.0628	1.0000	0.0712	0.0369	0.0235
Logistic Regression	0.0008	<0.0001	0.0023	0.0712	1.0000	0.0084	0.0041
TabTransformer	0.0894	0.1465	0.2081	0.0369	0.0084	1.0000	0.3012
TabNet	0.0527	0.1202	0.1806	0.0235	0.0041	0.3012	1.0000

**FIGURE 6:**
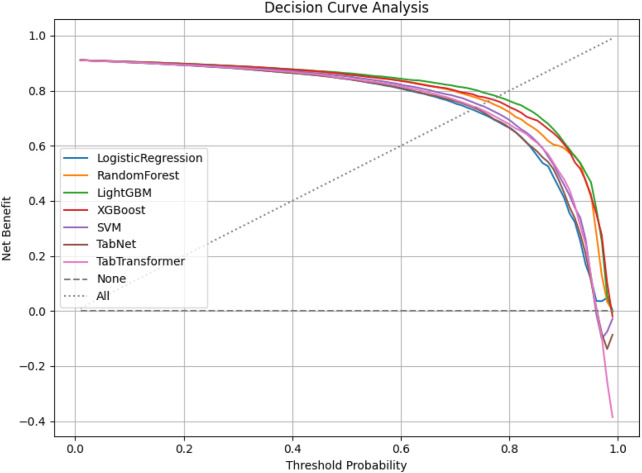
Decision curve analysis of all models.

Decision curve analysis (DCA) was conducted to assess the net clinical benefit of each model across a range of threshold probabilities. As shown in Figure 6, LightGBM and XGBoost demonstrated the highest net benefits, indicating superior clinical utility over both traditional and deep learning-based classifiers in high-risk chest pain prediction scenarios.

**FIGURE 7:**
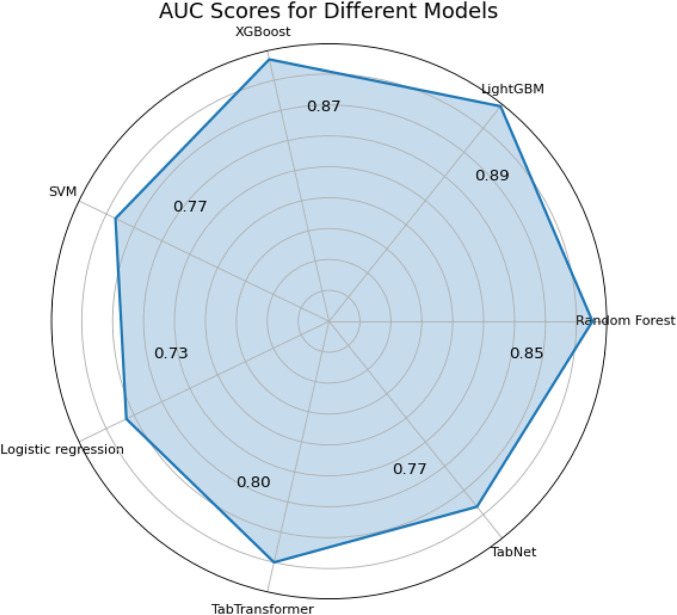
Radar map of ROC AUC assessment indicators.

**FIGURE 8:**
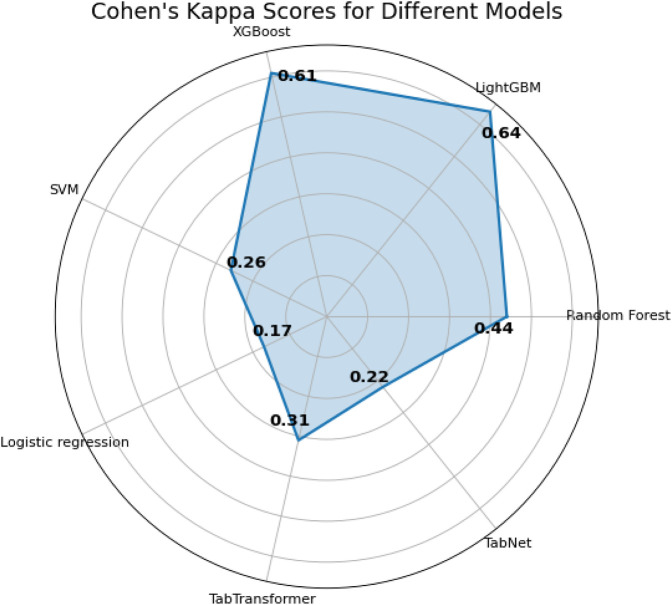
Radar map of Cohen’s Kappa (κ) assessment indicators.

The last two metrics used for model comparison are κ and ROC AUC scores (shown in Figure 7). Originally, κ was used to measure the level of agreement between two observers about a particular phenomenon, compensating for any agreement that might be caused by chance (Cohen, 1960). This ideology can also be applied to evaluate classification results. The results presented in Figure 7 show that both the LightGBM and XGBoost models belong to the better conformance category, with κ values of 0.64 and 0.61, respectively. In addition, the ROC AUC index is another evaluation metric for binary classification problems. The area under the curve (AUC) is used as a summary of the ROC curve, where larger areas are preferred (Ben-David, 2008). The results presented in Figure 8 show that both the LightGBM and XGBoost models belong to the superior category, with ROC AUC values of 0.89 and 0.87, respectively. Therefore, both κ and ROC AUC measurements are useful for validating the ability of each model to predict high-risk chest pain.

### Interpretability analysis

Global interpretability is essential for understanding the contribution of individual predictor properties to overall model performance. Accordingly, SHAP was employed to achieve global interpretation, with Shapley values used to assess feature importance in predictive outcomes. SHAP is a unified approach for explaining the output of any machine learning model, grounded in cooperative game theory. Specifically, it quantifies the marginal contribution of each feature to the prediction outcome of a given instance (Lundberg and Lee, 2017).

**FIGURE 9:**
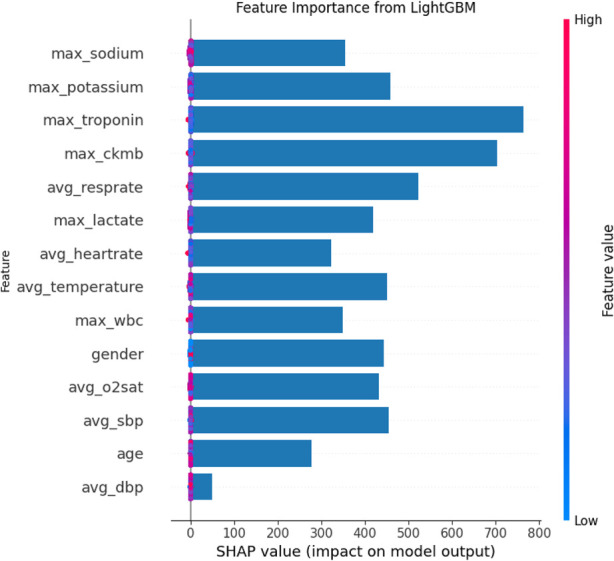
Shap analysis of the LightGBM model.

**FIGURE 10:**
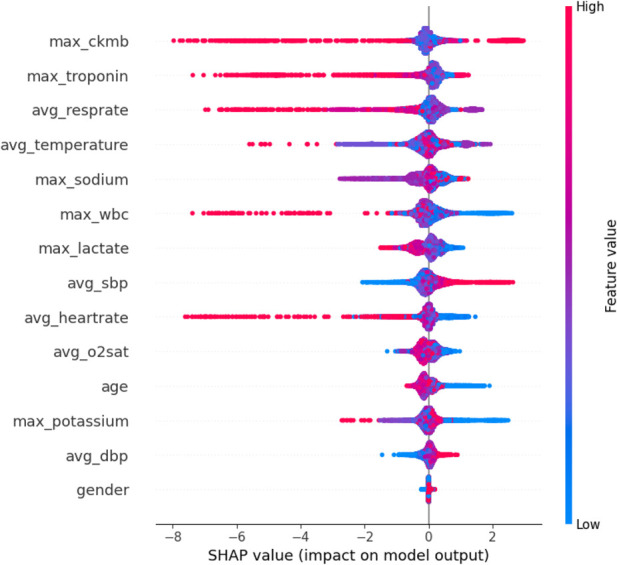
Global importance of LightGBM.

The SHAP feature importance bar chart is plotted using the average absolute Shapley value for each feature, where features with larger absolute Shapley values have higher priority. While this graph is useful, it does not provide any information other than a ranking of features based on importance (Joseph et al., 2022). Instead, the SHAP summary graph integrates feature importance with feature effects, where each point on the graph represents the Shapley value for each feature under that instance. The position on the y-axis determines the importance of the feature, while the Shapley value is located on the x-axis. The color of each instance represents the value of the feature, ranging from low (light blue) to high (pink). The scatterplot of SHAP values and the feature importance diagram for the LightGBM model are shown in Figures 9, 10.

As can be seen in Figures 9, 10, maximum troponin and maximum creatine kinase enzyme MB (CK-MB) are among the most influential features, with wide SHAP distributions. These biomarkers are medically validated indicators of cardiac injury and play a crucial role in the diagnosis of acute myocardial infarction (AMI). In fact, the 2023 European Society of Cardiology (ESC) guidelines emphasize high-sensitivity cardiac troponin as a key diagnostic tool for acute coronary syndromes (Collet, 2023), while CK-MB remains an established component of diagnostic criteria in many institutions (Alhusseini et al., 2024). The identification of these characteristics by the model as high impact supports its clinical relevance and interpretability.

In addition to cardiac biomarkers, features such as average respiratory rate, average body temperature, maximum sodium concentration, and maximum white blood cell count also exhibit strong SHAP values. These variables are frequently associated with systemic inflammation, infection, or metabolic imbalance, which are important secondary indicators of cardiovascular risk or severity of illness. In contrast, features such as sex and average diastolic blood pressure were assigned relatively low SHAP values, suggesting a limited impact on model predictions in this data set. Although gender is a known risk factor in the epidemiology of cardiovascular disease, its predictive value can vary depending on population balance, comorbidities, or feature interactions.

Overall, the SHAP analysis highlights that our model not only performs well but also aligns with established medical understanding. This reinforces trust in the model’s predictions and provides valuable insights for clinical interpretation, risk stratification, and future feature selection.

## Conclusions and future work

### Major contribution

Based on the MIMIC-IV database, this study proposed a feature engineering construction method for predicting high-risk chest pain and verified it by combining machine learning and deep learning models. Specific contributions include the following aspects:

1. Innovative construction of feature engineering. Through in-depth analysis of the MIMIC-IV data, a series of effective clinical features were designed and constructed, including physiological parameters, laboratory test results, basic patient information, etc. Efforts were made to uncover key factors that have a significant impact on the prediction of chest pain risk.
2. Experiment combined with BO algorithm. The Bayesian optimization algorithm was applied to optimize the hyperparameters of the model. Various machine learning models (such as Random Forest, XGBoost) and deep learning models (such as TabNet) were employed to conduct comparative experiments, demonstrating the performance of different models in predicting high-risk chest pain. The results showed that LightGBM achieved the best predictive performance, with an accuracy of 0.95, precision of 0.95, recall of 0.95, and F1 score of 0.94.
3. SHAP to achieve global interpretability. To improve model transparency and interpretability, the SHAP method was used to analyze the global interpretability of the prediction results. Analysis of SHAP values revealed the key factors influencing the model’s prediction of high-risk chest pain, thereby enhancing clinicians’ trust in the model.

### Limitations

Despite the promising results, this study has several limitations that warrant consideration:

1. Single-center data and lack of external validation. This study utilized retrospective data exclusively from the MIMIC-IV database, which may limit the generalizability of the model to other patient populations and clinical settings. External validation on independent cohorts, such as the eICU database or data from other institutions, is essential to assess robustness and broader applicability.
2. Simplified feature construction and imputation strategy. Clinical variables were summarized using static statistics (e.g., maximum, mean), potentially overlooking important temporal patterns. Additionally, missing values—including sensitive biomarkers like troponin—were imputed using regression-based methods, which may not preserve clinical plausibility and could introduce bias. Future work should incorporate time-aware modeling and clinically guided, distribution-preserving imputation techniques.
3. Limited interpretability and outcome label granularity. While global interpretability was addressed using SHAP, individual-level explanations were not explored, which limits clinical transparency. Moreover, the outcome definition of “high-risk chest pain” was based on retrospective labeling and may not capture the full clinical nuance of diagnostic decision-making. Prospective validation and expert-adjudicated labels are needed to enhance clinical relevance and trust.

### Future research direction

Although this study has achieved preliminary results in the task of predicting high-risk chest pain, there are still some limitations, which can be improved and expanded in the future from the following directions:

1. In-depth feature analysis and feature interaction exploration. Basic feature selection and construction were carried out on the MIMIC-IV dataset in this study, but the complex interactive relationship between features was not deeply explored. In the future, more advanced feature engineering methods, such as graph model-based feature interaction analysis or automated feature selection algorithms, can be used to explore nonlinear interactions between features and further improve the predictive power of the model. For example, Xie et al. (2023) proposed a multi-dimensional feature interaction modeling method based on deep neural networks, which provides a new idea for further feature interaction exploration.
2. Improving missing value processing methods. Missing value processing in clinical data has always been a major challenge in data preprocessing. In the future, more accurate missing value interpolation techniques can be explored, especially for complex clinical data, such as generating missing values through deep learning techniques such as generative adversarial networks (GAN), or using multiple interpolation methods (MICE) and Bayesian networks to improve the processing of missing data and reduce the prediction bias caused by missing data. Recent studies have shown that generative adversarial networks (GANs) have achieved good results in missing value interpolation of medical data (Lee et al., 2022).
3. Expansion to other tabular data models. In addition to existing machine learning models and TabNet, other deep learning models for tabular data can be explored in the future. For example, models such as disjunctive Normal Formula (DNF-Net) and Neuro-agnostic Decision Integration (NODE) (Puri and Sahoo, 2020), which have the potential to capture complex patterns in tabular data, can provide new ideas for high-risk chest pain prediction. Related studies, such as Zhang et al. (2024), proposed a clinical data analysis model based on NODE, and the experimental verification of this method on multiple datasets shows its powerful modeling ability.

## Data Availability

The data analyzed in this study is subject to the following licenses/restrictions: CITI. Requests to access these datasets should be directed to https://physionet.org/content/mimiciv/2.2/.
