# Supplementary material for: Machine learning prediction and interpretability analysis of high-risk chest pain: a study from the MIMIC-IV database
Source: Front Physiol. 2025 Jun 30;16:1594277. doi: 10.3389/fphys.2025.1594277 (PMC12256431; doi:10.3389/fphys.2025.1594277)

- Software：Navicat Premium 16.3.2
- Executive summary


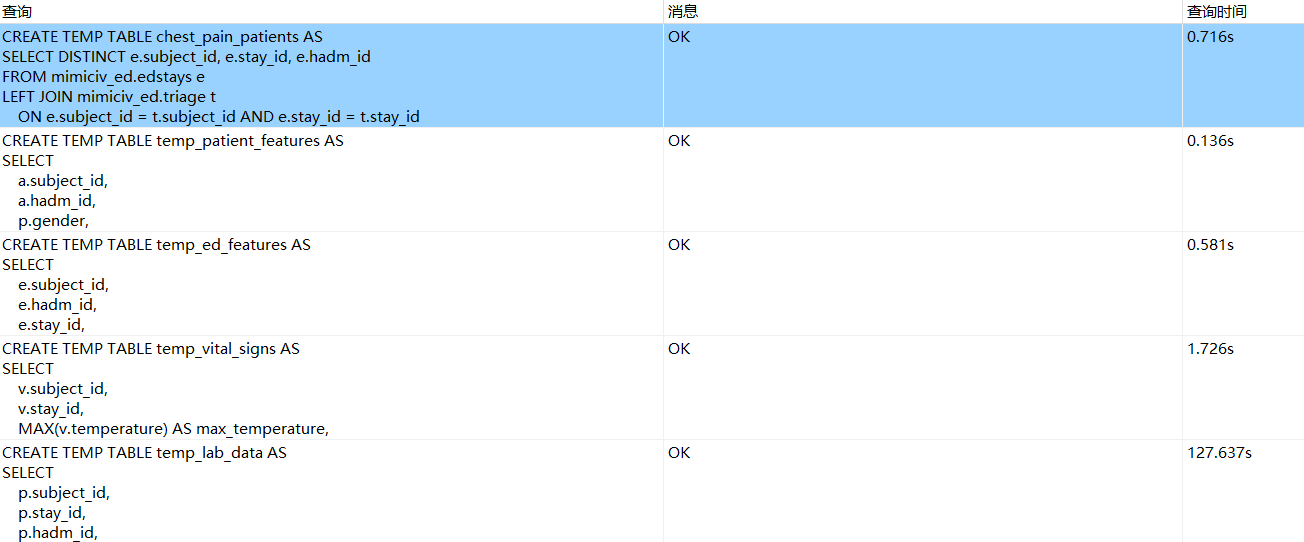


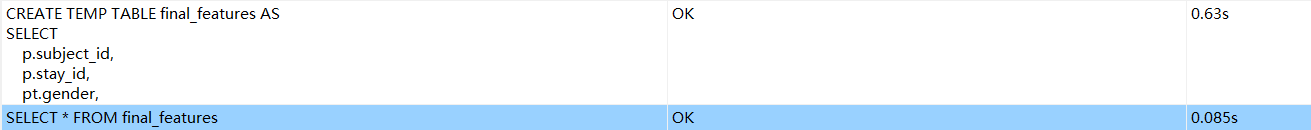


- **Code**

CREATE TEMP TABLE chest_pain_patients AS

SELECT DISTINCT e.subject_id, e.stay_id, e.hadm_id

FROM mimiciv_ed.edstays e

LEFT JOIN mimiciv_ed.triage t

ON e.subject_id = t.subject_id AND e.stay_id = t.stay_id

LEFT JOIN mimiciv_ed.diagnosis d

ON e.stay_id = d.stay_id

WHERE LOWER(t.chiefcomplaint) LIKE '%chest pain%'

OR LOWER(t.chiefcomplaint) LIKE '%angina%'

OR LOWER(t.chiefcomplaint) LIKE '%myocardial%'

OR d.icd_code IN ('78650', '78651', '78659', 'R071', 'R072', 'R079')

OR LOWER(d.icd_title) LIKE '%chest pain%'

OR LOWER(d.icd_title) LIKE '%angina%'

OR LOWER(d.icd_title) LIKE '%myocardial infarction%'

> OK

> 查询时间: 0.716s

CREATE TEMP TABLE temp_patient_features AS

SELECT

a.subject_id,

a.hadm_id,

p.gender,

p.anchor_age AS age,

p.anchor_year_group,

a.admittime,

a.dischtime,

DATE_PART('day', a.dischtime - a.admittime) AS hosp_length_of_stay,

a.admission_type,

a.hospital_expire_flag

FROM mimiciv_hosp.admissions a

JOIN mimiciv_hosp.patients p

ON a.subject_id = p.subject_id

WHERE a.hadm_id IN (SELECT DISTINCT hadm_id FROM chest_pain_patients)

> OK

> 查询时间: 0.136s

CREATE TEMP TABLE temp_ed_features AS

SELECT

e.subject_id,

e.hadm_id,

e.stay_id,

DATE_PART('minute', e.outtime - e.intime) AS ed_stay_length,

t.acuity AS triage_acuity,

t.chiefcomplaint,

STRING_AGG(m.name, ', ') AS medications

FROM mimiciv_ed.edstays e

LEFT JOIN mimiciv_ed.triage t

ON e.subject_id = t.subject_id AND e.stay_id = t.stay_id

LEFT JOIN (

SELECT

m.subject_id,

m.stay_id,

m.name

FROM mimiciv_ed.medrecon m

WHERE m.name IS NOT NULL

) m

ON e.subject_id = m.subject_id AND e.stay_id = m.stay_id

WHERE e.stay_id IN (SELECT stay_id FROM chest_pain_patients)

GROUP BY e.subject_id, e.hadm_id, e.stay_id, t.acuity, t.chiefcomplaint

> OK

> 查询时间: 0.581s

CREATE TEMP TABLE temp_vital_signs AS

SELECT

v.subject_id,

v.stay_id,

MAX(v.temperature) AS max_temperature,

MIN(v.temperature) AS min_temperature,

AVG(v.temperature) AS avg_temperature,

MAX(v.heartrate) AS max_heartrate,

MIN(v.heartrate) AS min_heartrate,

AVG(v.heartrate) AS avg_heartrate,

MAX(v.resprate) AS max_resprate,

MIN(v.resprate) AS min_resprate,

AVG(v.resprate) AS avg_resprate,

MAX(v.o2sat) AS max_o2sat,

MIN(v.o2sat) AS min_o2sat,

AVG(v.o2sat) AS avg_o2sat,

MAX(v.sbp) AS max_sbp,

MIN(v.sbp) AS min_sbp,

AVG(v.sbp) AS avg_sbp,

MAX(v.dbp) AS max_dbp,

MIN(v.dbp) AS min_dbp,

AVG(v.dbp) AS avg_dbp

FROM mimiciv_ed.vitalsign v

GROUP BY v.subject_id, v.stay_id

> OK

> 查询时间: 1.726s

CREATE TEMP TABLE temp_lab_data AS

SELECT

p.subject_id,

p.stay_id,

p.hadm_id,

MAX(cm.troponin_t) AS max_troponin,

MAX(cm.ck_mb) AS max_ckmb,

MAX(ch.sodium) AS max_sodium,

MAX(ch.potassium) AS max_potassium,

MAX(cb.wbc) AS max_wbc,

MAX(infl.crp) AS max_crp,

MAX(bg.lactate) AS max_lactate

FROM chest_pain_patients p

LEFT JOIN mimiciv_derived.cardiac_marker cm

ON p.subject_id = cm.subject_id AND p.hadm_id = cm.hadm_id

LEFT JOIN mimiciv_derived.chemistry ch

ON p.subject_id = ch.subject_id AND p.hadm_id = ch.hadm_id

LEFT JOIN mimiciv_derived.complete_blood_count cb

ON p.subject_id = cb.subject_id AND p.hadm_id = cb.hadm_id

LEFT JOIN mimiciv_derived.inflammation infl

ON p.subject_id = infl.subject_id AND p.hadm_id = infl.hadm_id

LEFT JOIN mimiciv_derived.bg bg

ON p.subject_id = bg.subject_id AND p.hadm_id = bg.hadm_id

GROUP BY p.subject_id, p.stay_id, p.hadm_id

> OK

> 查询时间: 127.637s

CREATE TEMP TABLE final_features AS

SELECT

p.subject_id,

p.stay_id,

pt.gender,

pt.age,

pt.anchor_year_group,

pt.hosp_length_of_stay,

pt.admission_type,

pt.hospital_expire_flag,

ed.ed_stay_length,

ed.triage_acuity,

CASE

WHEN ed.triage_acuity = 1 THEN 'High'

WHEN ed.triage_acuity IN (2, 3) THEN 'Medium'

WHEN ed.triage_acuity >= 4 THEN 'Low'

ELSE 'Unknown'

END AS risk_stratification,

ed.chiefcomplaint,

ed.medications,

vs.max_temperature,

vs.min_temperature,

vs.avg_temperature,

vs.max_heartrate,

vs.min_heartrate,

vs.avg_heartrate,

vs.max_resprate,

vs.min_resprate,

vs.avg_resprate,

vs.max_o2sat,

vs.min_o2sat,

vs.avg_o2sat,

vs.max_sbp,

vs.min_sbp,

vs.avg_sbp,

vs.max_dbp,

vs.min_dbp,

vs.avg_dbp,

lab.max_troponin,

lab.max_ckmb,

lab.max_sodium,

lab.max_potassium,

lab.max_wbc,

lab.max_crp,

lab.max_lactate

FROM chest_pain_patients p

LEFT JOIN temp_patient_features pt

ON p.subject_id = pt.subject_id AND p.hadm_id = pt.hadm_id

LEFT JOIN temp_ed_features ed

ON p.subject_id = ed.subject_id AND p.stay_id = ed.stay_id

LEFT JOIN temp_vital_signs vs

ON p.subject_id = vs.subject_id AND p.stay_id = vs.stay_id

LEFT JOIN temp_lab_data lab

ON p.subject_id = lab.subject_id AND p.stay_id = lab.stay_id

> OK

> 查询时间: 0.63s

SELECT * FROM final_features

> OK

> 查询时间: 0.085s

- Executing the success page


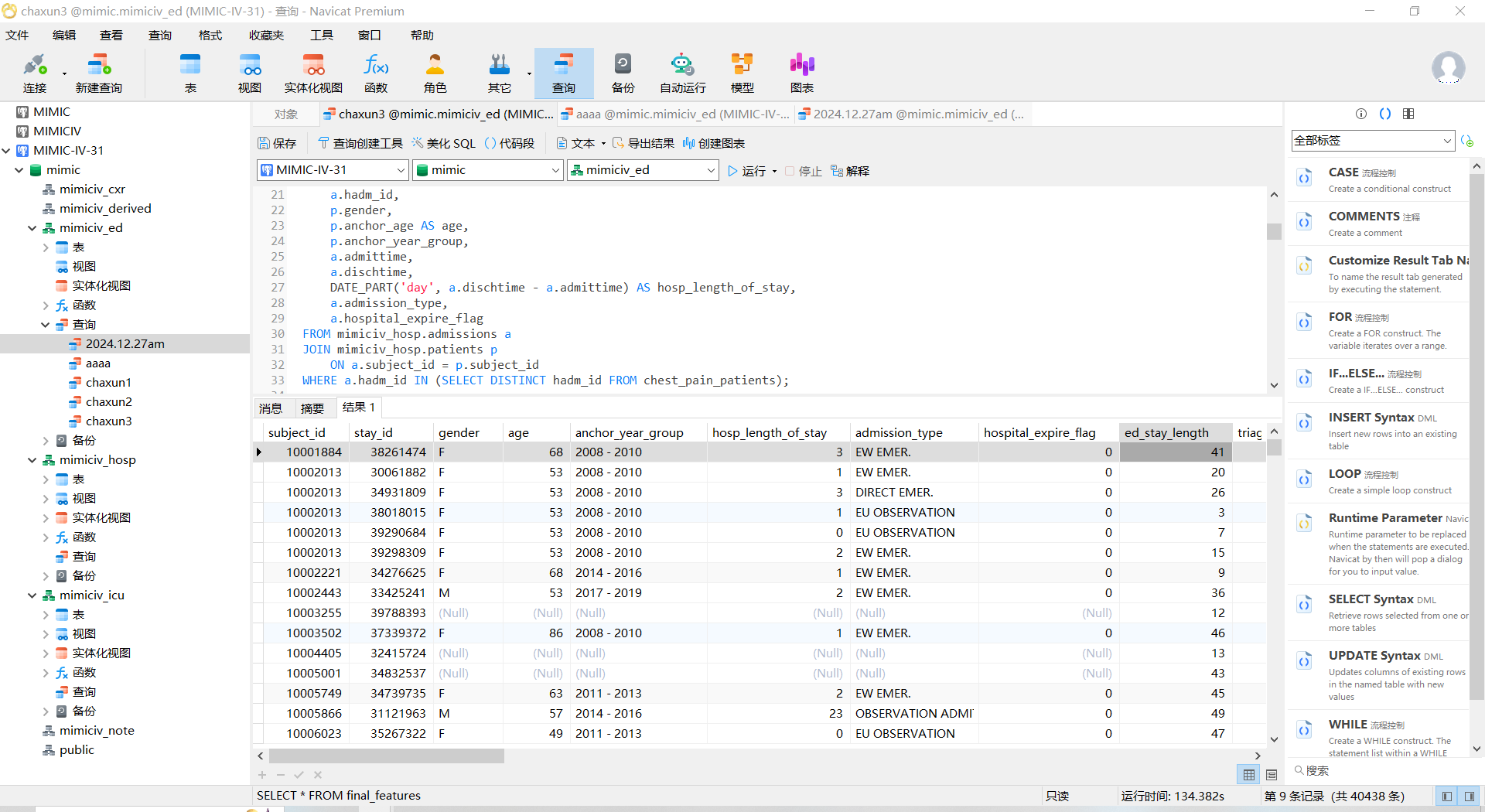

Supplement: Supplementary file 1 [file DataSheet1.zip › Supplementary Material/SQL Code.docx]
