# Supplementary figures and images for: Machine learning prediction and interpretability analysis of high-risk chest pain: a study from the MIMIC-IV database
Source: Front Physiol. 2025 Jun 30;16:1594277. doi: 10.3389/fphys.2025.1594277 (PMC12256431; doi:10.3389/fphys.2025.1594277)

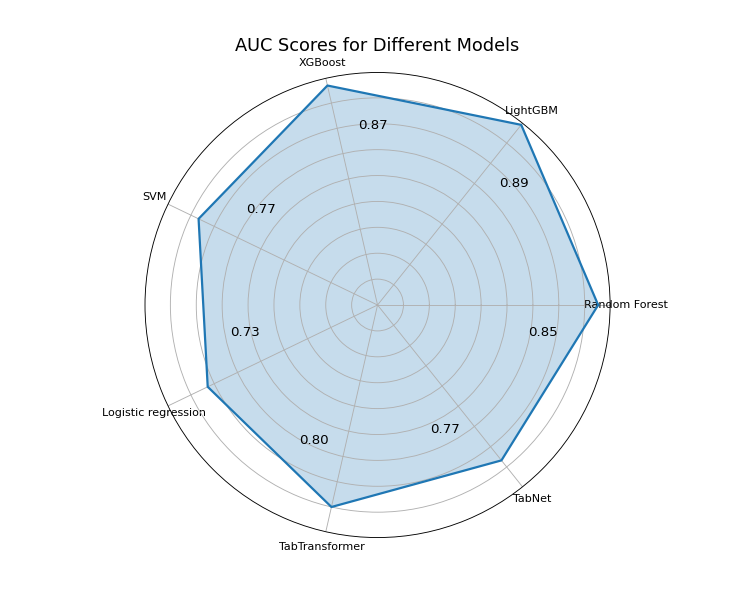

Supplement: Supplementary file 1 [file DataSheet1.zip › Supplementary Material/Figure/auc.png]

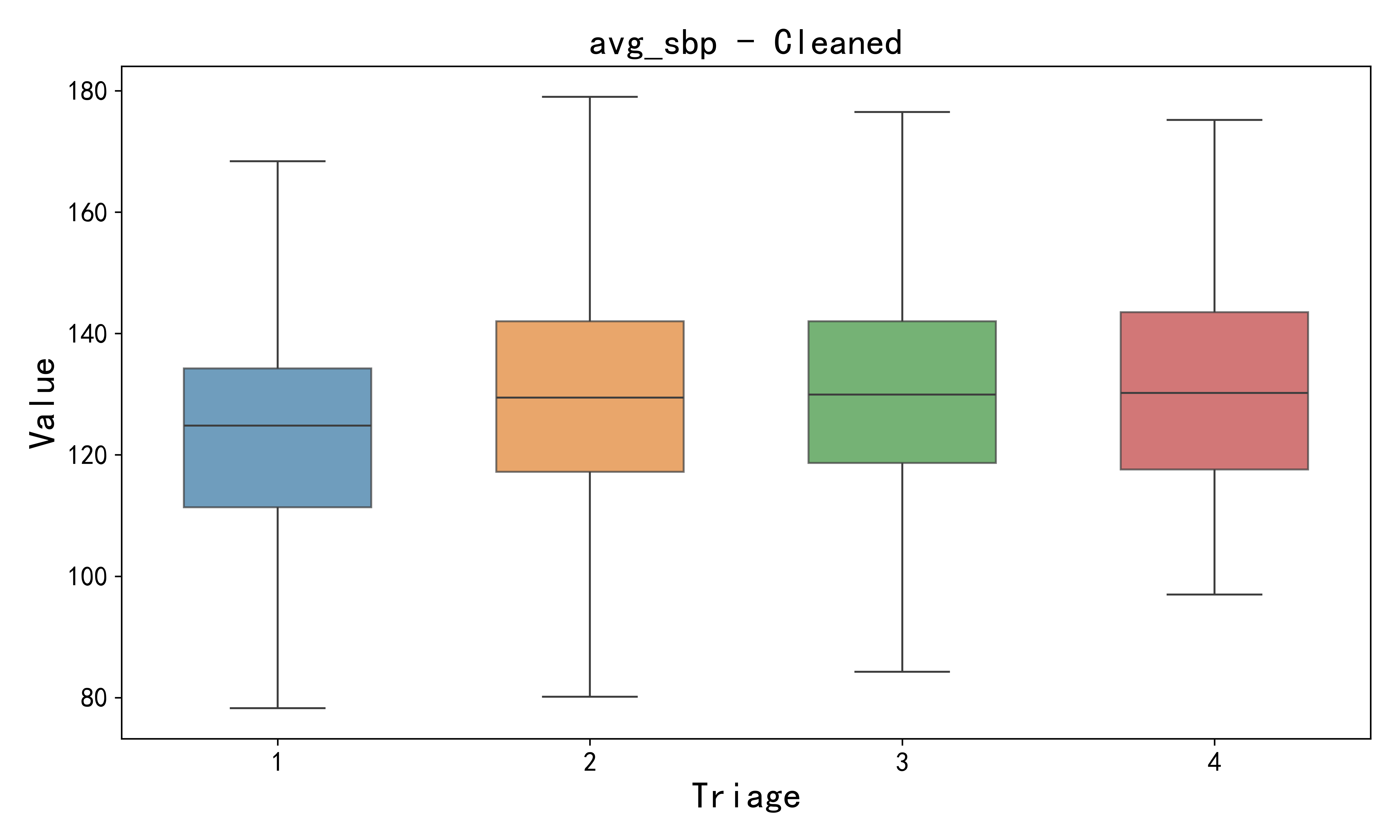

Supplement: Supplementary file 1 [file DataSheet1.zip › Supplementary Material/Figure/Cleaned.png]

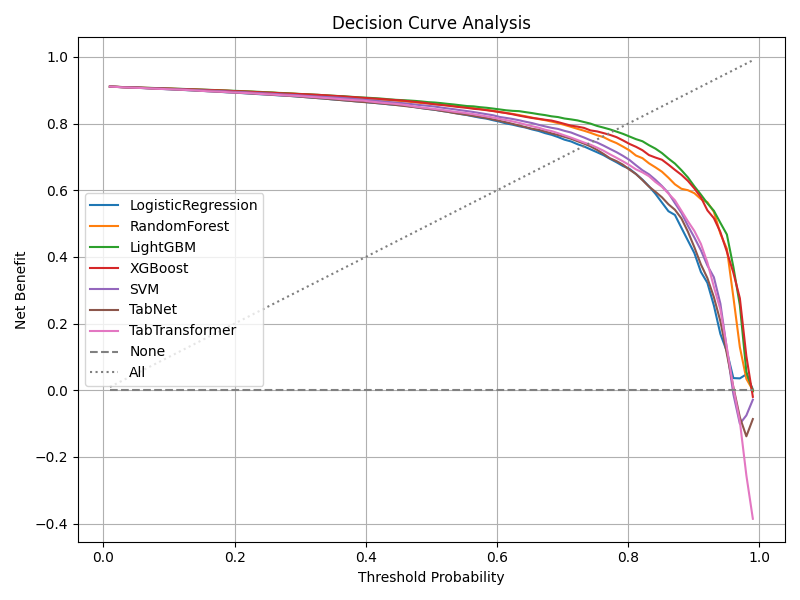

Supplement: Supplementary file 1 [file DataSheet1.zip › Supplementary Material/Figure/decision_curve.png]

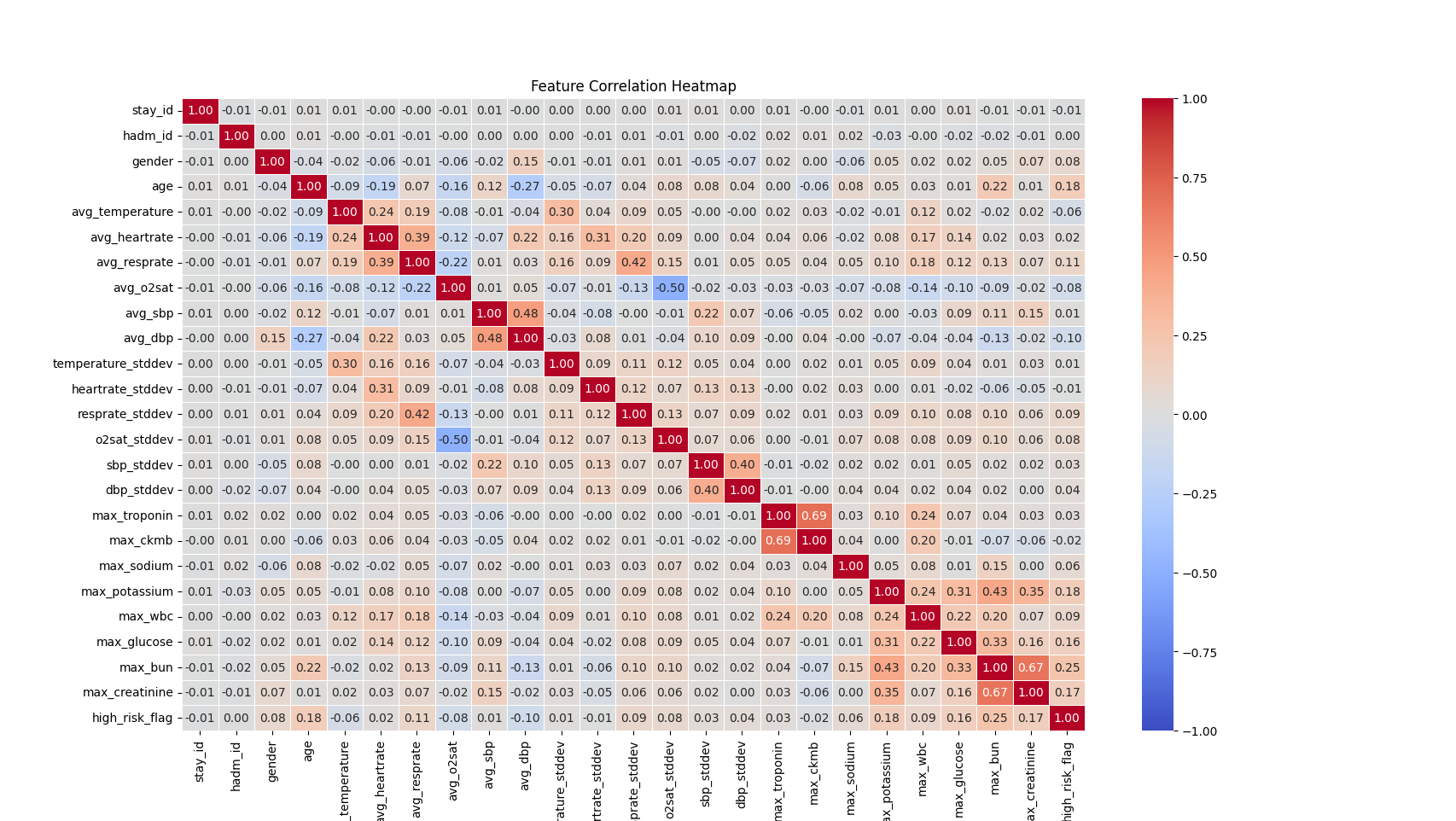

Supplement: Supplementary file 1 [file DataSheet1.zip › Supplementary Material/Figure/hot.png]

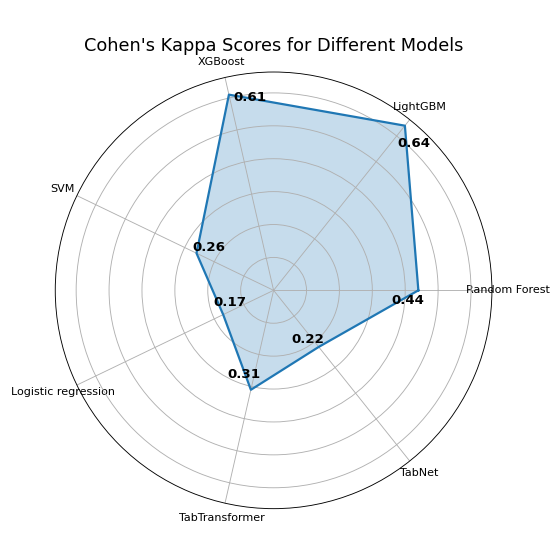

Supplement: Supplementary file 1 [file DataSheet1.zip › Supplementary Material/Figure/kappa.png]

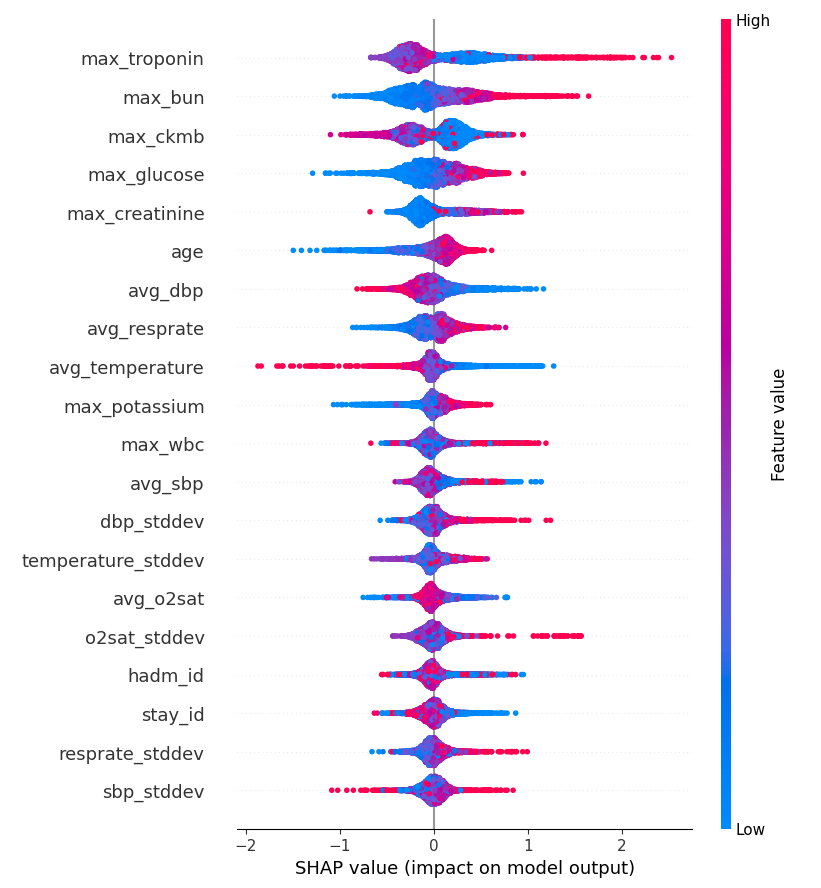

Supplement: Supplementary file 1 [file DataSheet1.zip › Supplementary Material/Figure/new.png]

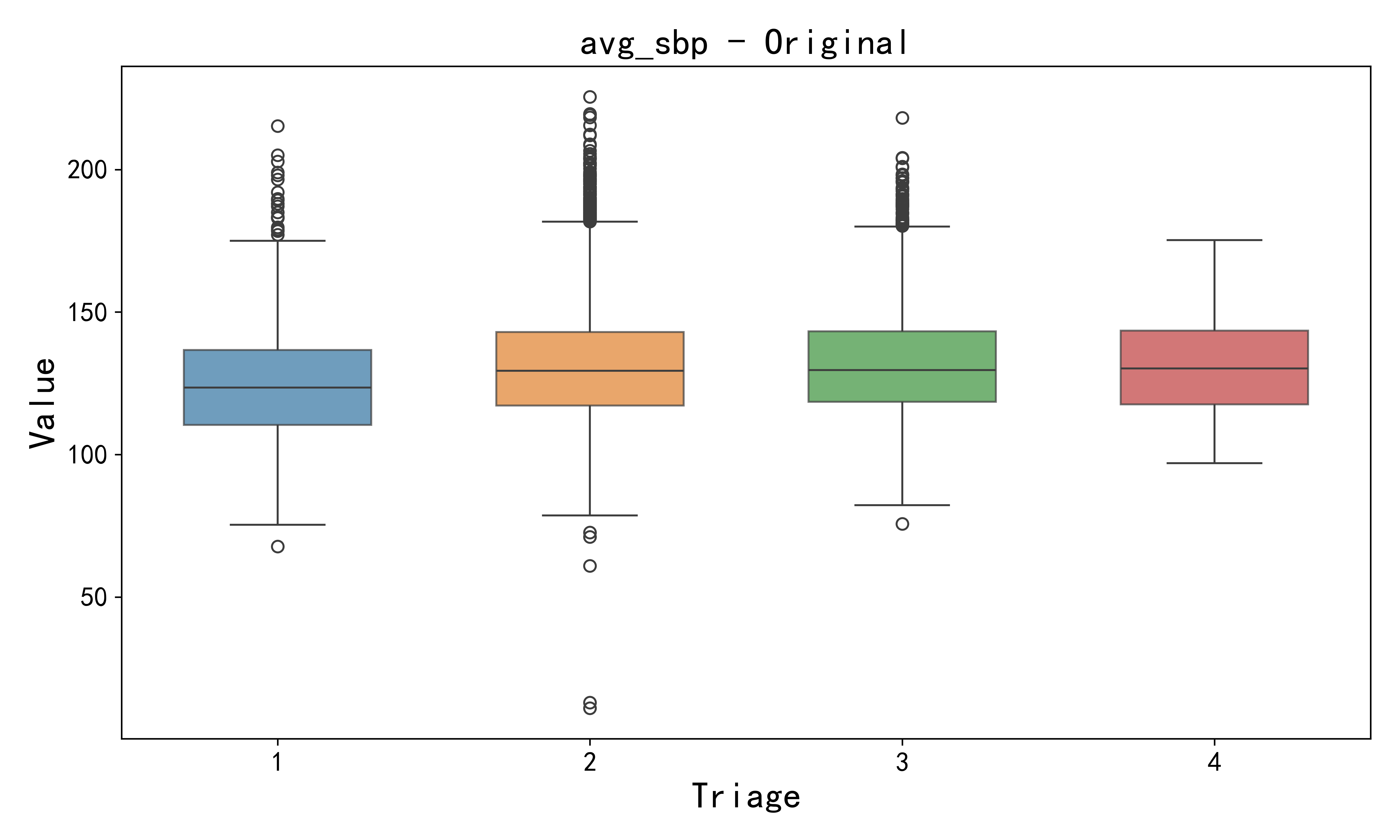

Supplement: Supplementary file 1 [file DataSheet1.zip › Supplementary Material/Figure/original.png]

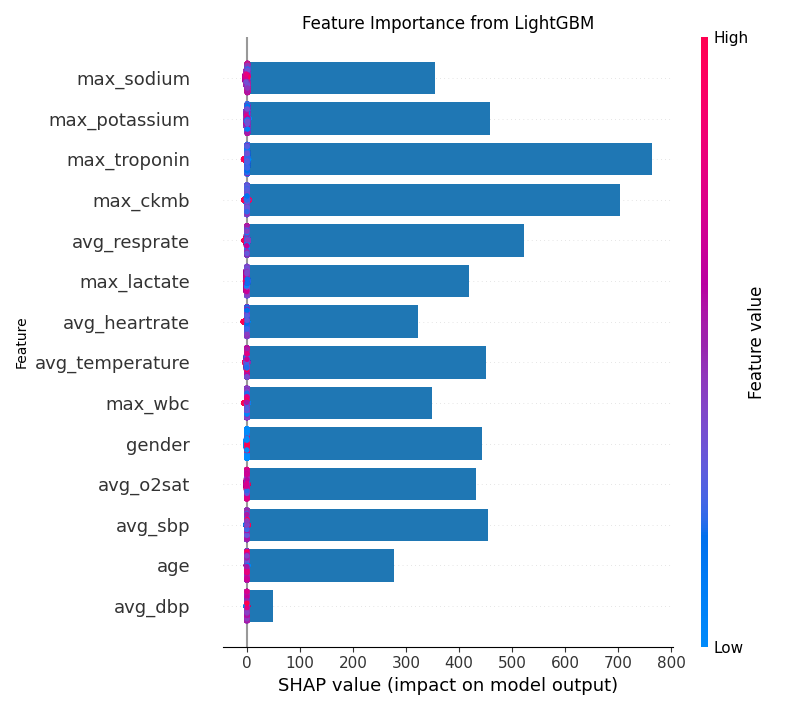

Supplement: Supplementary file 1 [file DataSheet1.zip › Supplementary Material/Figure/shap.png]
